# The effects of applying an augmented reality English teaching system on students’ STEAM learning perceptions and technology acceptance

**DOI:** 10.3389/fpsyg.2022.996162

**Published:** 2022-10-14

**Authors:** Yu-Sheng Su, Chieh-Chun Lai, Ting-Kai Wu, Chin-Feng Lai

**Affiliations:** ^1^Department of Computer Science and Engineering, National Taiwan Ocean University, Keelung City, Taiwan; ^2^Department of Engineering Science, National Cheng Kung University, Tainan City, Taiwan

**Keywords:** STEAM education, augmented reality, English course, technology acceptance, learning perception

## Abstract

With the inherently interdisciplinary and hands-on nature of the STEAM system, this study changes the way the English language is taught, no longer confining it to monotonous tests and dull textbooks. Teaching *via* Augmented Reality (AR) technology tends to pique students’ interest, making them more willing to participate in learning. This study used AR technology in conjunction with gaming, where the participants were split into pairs, which promoted friendly competition among themselves, and, in turn, drove them to learn more. Moreover, the ability to track their own performance was added to the system, as well as an in-depth explanation for every question. The teacher used our teaching aids system in the lessons to make the teaching process more interesting and enjoyable. There were two English course experiments, each lasting for 4 weeks. The goal of the first experiment was to gain a general understanding of how students felt about the lessons and how well they could operate the system. After analyzing the results, some improvements for problems that arose were made in the second experiment. The goal of the second experiment was to find out how students felt about the lesson content and teaching aids and to compare the results of the two experiments. To help us find potential oversights and check how participants felt about this approach, surveys regarding STEAM learning and overall ease of use were administered. The results showed that AR English learning was widely considered helpful by the students, and also, our system was easy to operate, and competing with peers really motivated them to do better.

## Introduction

As the term “World Village” becomes increasingly prevalent, it is crucial to keep up with the rest of the world. How can one better connect with the world? English is the key, as it is the most widely used language, making it one of the best ways to connect with the rest of the world. English learning has been a staple in the Taiwanese education system for some time now, further proving its importance, at least in Taiwan, as a tool to communicate globally. “An uncut gem does not sparkle” is a sentiment that many parents and teachers share, which stems from the more traditional and rigid ways of teaching. However, those methods of education are now looked upon as monotonous and standardized to a fault, which might inadvertently suppress the students’ creativity and willingness to study (Li, 2017).

Following the realization that applying what our study has taught us is critical, STEAM is starting to see a rise in popularity, as it is one of the best ways to teach students how to actually put what they have learned into practice. Combining Science, Technology, Engineering, Art, and Mathematics, STEAM is an interdisciplinary method of teaching, making it more interesting for students to engage in the learning process (Yakman and Lee, 2012; Chung et al., 2020).

Chung et al. (2020) designed a course in STEAM education in 2020 and hosted a design competition for pet wearable devices. They split the students into groups and had them solve problems relevant to the theme. Students needed to design their own wearable pet devices for realistic purposes using interdisciplinary knowledge. Constant exchange of ideas and reflection between them and the teachers piqued students’ learning interests.

English has now become one of the necessary abilities for connecting with the world, and this study built upon the framework of STEAM, combining it with Augmented Reality (AR) technology to integrate gaming into the learning of English. As stated above, we proposed two research questions.

• How do the students feel when they use the Augmented Reality English Teaching System?
• What is the students’ technology acceptance of using the Augmented Reality English Teaching System?

## Literature review

STEAM is not only about multidisciplinary learning and applying knowledge to everyday events, but also about the dynamics between student and teacher. By learning through feedback, students are more willing to deepen and broaden their learning, as well as become interested in acquiring new knowledge (Yakman and Lee, 2012; Chung et al., 2020). Tytarenko et al. (2021) applied the STEAM framework to English education and ran an experiment involving students. The students were split into two groups, one group taught normally and the other with the new framework. Over the course of half a term, it was clear that students taught under the STEAM system yielded better results, improving more than the other group in all four aspects of the English language.

Differing from prior methods of teaching, the biggest hurdle to overcome while following STEAM is to come up with courseware, as to fulfill interdisciplinary learning means that the teacher must be familiar with multiple subjects. Yakman and Lee (2012) stated that teachers from various fields have to work together to ensure that students can truly understand the differences between domains. Tytarenko et al. (2021) also mentioned that properly carrying out the five aspects of STEAM and integrating them is crucial in this method of teaching. Weaving education into everyday life would be a great way to efficiently develop the students’ ability to put knowledge into practice, and STEAM at its core is about letting students learn from the action of “playing with toys,” teaching them while making it interesting and fun for them. Thus, integrating STEAM with “toys” is most definitely the way to weave in education in an enjoyable manner.

The most common toys for children of this generation are undoubtedly smartphones and tablets, as there are countless applications that capture their attention, and many of them are even educational. Makoe and Shandu (2018) designed an app for English vocabulary that makes use of a “daily vocabulary” feature that motivates users to come back every day, making it a habit in the process. As the world moves toward an era of digital entertainment, video games are now more abundant than ever, including educational video games. Chen et al. (2018) even took it a step further and implemented AR gaming into education, captivating children and motivating them to learn.

There have been several past instances of using AR or Mixed Reality (MR) technology in education. Previous studies (Hughes et al., 2005; Schrier, 2006; Ahn and Choi, 2015; Birt and Cowling, 2017; Chen et al., 2018) have shown that it is beneficial for students’ understanding, while also raising their motivation to study.

Chen et al. (2018) compared the effect of learning *via* video games to that of AR games, and their experiment showed that students who learned with AR games achieved better results and had stronger motivation. However, those AR games lacked answer explanations and the feature for students to access their learning records. There was also a lack of question quantity and variety, resulting in the inability of individuals to efficiently focus on their weaknesses. Hughes et al. (2005) explored how MR allows the magic of virtuality to escape the confines of the computer and enter our lives to potentially change the way we play, work, train, learn, and even shop.

As mentioned in Schrier’s (2006) research paper, the proper manipulation and combination of technical skills and knowledge can greatly enhance students’ motivation to learn. However, in Birt and Cowling’s (2017) research experiments, it was shown to be more difficult to combine MR and knowledge; especially if used as a tool for teaching, it could be rather inflexible. Direct means of combining MR and academic knowledge might be able to solve the various problems previously faced when planning for program activities and architecture. In addition, the use of games to impart knowledge can greatly diminish the difficulty of implementing MR into the teaching of academic knowledge. With the use of MR, teaching methods do not have to be changed according to the academic subjects but can be simply adapted to the games themselves. This can also promote flexibility in teaching those subjects, especially since there is a less steep learning curve, thus allowing users to use AR tools with less difficulty.

This study focused on using digital games combined with MR to raise learners’ curiosity and pique their interest. This is different from Chen et al.’s (2018) research, as this research used AR and in addition, leveraged STEAM educational theories to design the game. The approach chosen for the game is a two-player, player vs. player method, which not only creates fun and joy for learners but also creates a competitive spirit among them. Based on past experiments, new functionalities were also included, where learners could check on their status and progress in the midst of learning, which they could then review and reflect on, inculcating in them the learning spirit that STEAM wishes to achieve.

To verify that the application of STEAM in English is useful, this study referenced Griese et al.’s (2015) and Sun et al.’s (2021) questionnaires and designed a STEAM learning perceptions questionnaire. It provides numerous different ways of analyzing students’ concentration, organizational capacity, and peer interaction when using the STEAM English education system. So as to know how the students felt when they used the combination of AR technique and English, this study also referenced Chang’s questionnaire to design a questionnaire on the acceptance of technology. There are two dimensions to the questionnaire, namely Perceived ease of use, and Perceived usefulness.

## Methodology

### Participants

This study cooperated with teachers from North Taiwan in developing the English course experiment. We chose two equivalent classes in which the students were both seventh-graders, executing the English course experiment separately. Each of the experiments had 30 students participate, and a total of 60 students took part in this experiment activity. The teacher went through how to operate the Augmented Reality English Teaching System beforehand with the students, and any additional problems were resolved during the experiment to let students participate in English class through the Augmented Reality English Teaching System.

### Learning materials

The focus on common dialogues, basic grammar, and everyday vocabulary aimed to help students build conversational and comprehension skills. This study gathered experience and advice from junior high school teachers, which resulted in changing and improving our course material before finally setting the focus on tenses, vocabulary, and common phrases, at the same time using AR technology in conjunction with the STEAM education approach to develop this AR English Teaching System.

The learning materials mainly focused on the application of bionic bird technology and the different elements of STEAM. In the science element, students learned about common bird habits and habitats; in the technology element, they learned about bird behavioral bionic technology and integrated it into English conversations; in the engineering element, this study presented real-life applications of bionic bird technology, such as trains and airplanes, through the reading and dialogue questions in the system. In the art element, with their imagination and the knowledge they had gained up to this point, students made their own paper planes. Last, in the mathematics element, students learned to give descriptions of aspects such as body length, flight distance, and flight altitude in English, and then measured their paper planes and flights.

### Procedure

We performed this experiment with students in a junior high school located in northern Taiwan to find out what students would feel about STEAM education and learning with technology after using the Augmented Reality English Teaching System. The experiment lasted for 4 weeks, during which there were two English course experiments. The goal for the first one was to gain a general understanding of how students felt about the lessons and how well they could operate the Augmented Reality English Teaching System, and after analyzing the results, they developed solutions for any problems that arose. The second experiment aimed to find out how students felt after improvements were made to the lesson content and teaching aids, and compared the results of both experiments after analyzing the data.

The teacher taught the class, as usual, only using the Augmented Reality English Teaching System as a supplementary tool for the lessons, all of which were 80 min long. During the first week, before each lesson, the teacher explained thoroughly how the experiment would work, the rules and goals of the experiment, and how to operate the Augmented Reality English Teaching System. The second and third weeks were when the experiment actually took place, making every lesson 80 min long. The second week focused on vocabulary, grammar, and basic sentence structures for describing birds, whereas the third week integrated knowledge about bionic bird technology, building upon the content from the previous week. The teacher used the Augmented Reality English Teaching System as a supplementary tool for the lessons, splitting the students into groups of two and monitoring their progress through the server. The fourth and final week was when students made use of all they had learned in the previous weeks to design and build their own paper planes. They were also encouraged to go on stage to share their designs with the class. After everything was finished, the teacher conducted surveys on how students felt about the STEAM education system, as well as how they felt about learning with the new technology. The data that were collected throughout the whole process were then analyzed.

### Instruments

#### Augmented reality English teaching system

The Augmented Reality English Teaching System can be split into the English learning app (student client), the teacher’s side (teacher client), servers, user database, questions database, and courseware database. Teacher client users can upload questions to the courseware database through the server, and choose questions from the courseware database to put into the questions database. After students register, they can log into the English learning app, and the server then picks questions from the questions database for the student to answer. After answering, the server uploads the results to the user database, and students can know at any point if their answers were right or wrong, as well as get in-depth explanations of the questions. Teachers can also check on their students’ progress through the server. This study merged the Augmented Reality English Teaching System with other subjects by making a 1 × 1 competitive game, using smartphones, tablets, and other devices to answer multi-choice questions. Making use of everyday devices motivates the students to study, while the flexibility that the Augmented Reality English Teaching System brings to the table by letting the teacher have control over the courseware, along with the friendly competition inherent within competitive games all contribute to raising the efficiency with which students can learn English. This research also documented data on students’ learning progress and analyzed their performance, tabulating the data immediately after each question. Students could then check on their own statistics and work on their flaws. The teacher could understand students’ abilities and focus on the areas that they were weak at, thus boosting their future performance.

#### STEAM learning perceptions questionnaire

The questionnaire regarding STEAM learning was designed with reference to Griese et al.’s (2015) and Sun et al.’s (2021) questionnaire designs and was split into six categories: Monitoring, Organization, Repeating, Elaboration, Attention, and Peer Learning, with a total of 35 questions, to determine the level of understanding students have of STEAM and English in general. The 5-point Likert scale corresponded to a score of 1 through 5, with Strongly Agree correlating to 5.

• Monitoring: The level of understanding students have of the content of the lessons.
• Organization: The students’ ability to summarize and grasp the key takeaways of each lesson, helping them memorize it.
• Repeating: How many students do repeat exercises to help memorize vocabulary and grammar?
• Elaboration: The level of effectiveness with which students can apply their knowledge in real life.
• Attention: The level of focus that students can maintain in class.
• Peer Learning: The level of competence that students show when working with their peers to solve problems.

#### Technology acceptance questionnaire

The questionnaire took heavy inspiration from Chang et al.’s (2011) design, splitting the total of nine questions into two sections: five questions for Perceived Usefulness, and four questions for Perceived Ease of Use. The main purpose of this questionnaire was to find out the level of acceptance students had for using Augmented Reality technology in class. The questionnaire makes use of the 5-point Likert scale, with Strongly Agree correlating to 5.

• Perceived Usefulness: How useful do students think the system is for helping them improve their English?
• Perceived Ease of Use: How difficult do students think it is to operate the system?

## Results and discussion

### Results of the first English lesson

In our first experiment, the average scores for the STEAM Learning Impressions questionnaire were as follows: 3.583 for Monitoring, 3.370 for Organization, 3.120 for Repeating, 2.983 for Elaboration, 3.234 for Attention, and 3.280 for Peer Learning. The averages of the Technology Acceptance questionnaire were 3.000 for Perceived Usefulness, and 4.217 for Perceived Ease of Use. These statistics show that the system designed for this study not only lowered the difficulty for students to operate AR teaching but also resolved the issue where students could not check on their progress and learn through feedback. The study was not without its flaws, however, as there were problems where if the paired-up students differed too much in terms of their skill level, their overall experiences would be lackluster. Another issue the study struggled with was the connection between classes and everyday life. It is speculated that students living in a Chinese-speaking environment are the reason behind this, as it is difficult for them to digest and apply their English learning when they barely use it in real-life scenarios. To address these issues, an effort was made to pair up students whose proficiency in English was around the same level to avoid the potential dull learning experience that comes with the discrepancy between student abilities, while also working with the teachers to condense the course down to the aforementioned common sentences, tense grammar, and everyday vocabulary, to give students an easier time grasping the main takeaways of each lesson, and applying those takeaways to their lives. The following are the results of the second experiment.

### Results of the second English lesson

• How do the students feel when they use the Augmented Reality English Teaching System?

The main purpose of the STEAM Learning Perceptions questionnaire was to determine the level of understanding that students had of STEAM and English when using the Augmented Reality English Teaching System. The questionnaire consists of six different dimensions: Monitoring, Organization, Repeating, Elaboration, Attention, and Peer Learning, with a total of 35 questions. This study used the one-sample *t*-test on the average scores of 3.583, 3.370, 3.120, 2.983, 3.234, and 3.280 for each of the respective dimensions, and the results were: Monitoring (Mean = 4.122, S.D. = 0.703, *t* = 4.201, *p*-value < 0.001), Organization (Mean = 3.967, S.D. = 0.816, *t* = 4.003, *p*-value < 0.001), Repeating (Mean = 4.153, S.D. = 0.750, *t* = 7.551, *p*-value < 0.001), Elaboration (Mean = 4.044, S.D. = 0.665, *t* = 8.739, *p*-value < 0.001), Attention (Mean = 3.917, S.D. = 0.920, *t* = 4.065, *p*-value < 0.001), and Peer Learning (Mean = 4.040, S.D. = 0.729, *t* = 5.714, *p*-value < 0.001). Table 1 shows the results of each dimension of the STEAM Learning Perceptions questionnaire. Of the 35 questions, 21 had an average score higher than 4, and the other 14 had averages higher than 3. This shows that the majority of the students agreed that using the Augmented Reality English Teaching System was helpful for learning English and that they generally had a good experience making charts, focusing on the class, and learning with peers, among other activities, meaning that the system was well received.

**TABLE 1:** Results of the STEAM learning perceptions questionnaire.

Item	N	Mean	S.D.
**Monitoring dimension**			
I can properly identify and explain the main takeaways of every unit.	30	4.200	0.714
I can spell out the important vocabulary from each lesson without a problem.	30	4.233	0.858
I can pronounce the important vocabulary from each lesson without a problem.	30	4.133	0.860
I can use the vocabulary in sentences without a problem.	30	4.100	0.845
I can properly understand English sentences.	30	4.200	0.847
I ask myself grammatical problems to make sure that I understand everything in the lessons.	30	3.867	1.042
**Organization dimension**			
I group words of similar meaning together.	30	4.067	0.944
I organize and memorize relevant points in the lessons	30	4.033	0.890
I organize and memorize grammatical rules.	30	3.933	1.015
I use graphs to help me memorize the contents of each unit.	30	3.700	1.149
I use graphs to help me understand the differences between grammatical rules.	30	3.867	1.196
I am able to make a basic chart about the lesson topics.	30	3.933	1.081
I can properly grasp the key points in each unit.	30	4.233	0.774
**Repeating dimension**			
I memorize vocabulary by writing it down repeatedly.	30	4.000	0.947
Through continuous practice on similar questions, I am able to understand the logic behind each question.	30	4.333	0.884
I memorize vocabulary by repeatedly using the word in different sentences.	30	4.000	0.910
Repeated exercises help me remember the contents of a lesson.	30	4.167	0.913
Continuous reading of English paragraphs help improve my sense of language.	30	4.267	0.980
**Elaboration dimension**			
I am able to use what I learned in class in practice.	30	4.167	0.834
I can think of ways to use new concepts learned in class in practice.	30	4.067	0.907
I often try using English to say something I have said in Chinese.	30	4.067	0.828
I regularly think about how to use what I learned in class in practice.	30	3.933	0.868
I would like to know if the lesson content is applicable in real life.	30	4.133	0.860
I am able to explain the key points of each unit and how they are connected.	30	3.900	0.960
**Attention dimension**			
I am able to stay focused during English class.	30	3.967	0.964
I don’t get easily distracted during English class.	30	4.033	1.033
I don’t do unrelated things when studying English.	30	3.967	0.964
I don’t get easily distracted by events and things around me when studying English.	30	3.967	1.066
I am able to fully concentrate on the English lesson.	30	3.867	1.074
I am able to stay focused for long periods of time when studying English.	30	3.700	1.119
**Peer learning dimension**			
I can explain the main point of the English course, and make sure that other students understand.	30	3.867	1.042
When I can’t comprehend the content of the English course, I take the initiative to discuss it with other students.	30	4.000	0.910
Learning English with my classmates motivates me more.	30	4.133	0.900
If another student performs better than me, it can motivate me to study English.	30	4.000	0.910
Q&A sessions on the English course contents with classmates can help me make progress in learning English.	30	4.200	0.887

Monitoring had a higher average score than other dimensions (Mean = 4.1222), and also showed a huge improvement from the first experiment (Mean = 3.583). The first five questions in Table 1 had an average score higher than 4, with question 2 having the highest (Mean = 4.233), indicating that students were able to spell out vocabulary in the courseware. It also shows that most students had a good understanding of the lesson content. Students could identify key points in each unit, make sentences with taught vocabulary, and even understand the grammatical rules. This result is due to refining the course material down to only a couple of units, which helped students identify key points easily. However, question 6 had considerably worse results (Mean = 3.867, S.D. = 1.042), and we speculate that rather than asking themselves questions to make sure of their understanding of the lesson, some students only did it through tests and homework.

The average score for the Organization dimension was 3.966, a considerably higher score than the 3.370 in the first experiment, and it can also be seen that question 7 in the Organization dimension in Table 1 had the highest average score (Mean = 4.233), meaning that most students were able to recognize the key points of the lesson. The other questions all had a score close to 4, which shows that the majority of the students made charts to help organize and memorize the lesson content, and compared different grammatical rules. These scores also show that refining the lesson content makes it easier for students to summarize each unit and organize similar points.

The Repeating dimension had an average score higher than 4 (Mean = 4.1533), and it was also higher than that of the first experiment (Mean = 3.120). It is speculated that as a result of refining the course material, users of the Augmented Reality English Teaching System could now do more repeated practices than before, which is also why question 2 in the Repeating dimension in Table 1 boasted a considerably higher average score than the others (Mean = 4.333), signifying that repeated practice helps students memorize important content. All other questions in the Repeating dimension also had a score higher than 4, which shows that repeated practice not only helped the majority of students learn more efficiently but also helped strengthen their sense of language. It is believed that this is also part of the reason behind the higher averages in the Monitoring dimension.

The average score for the Elaboration dimension was 4.044, and as seen in the Elaboration dimension in Table 1, question 1 had the highest average score (Mean = 4.167), indicating that most students could put knowledge into practice, turning what they had learned in class into tools of their own. Question 5 also had an average score higher than 4, indicating that most students wanted to know the relation between the lesson content and real life. The other questions all had an average of somewhere around 4, which highlights the importance of gaining knowledge and applying it to real life. However, with the Elaboration dimension in the first experiment having an average score of 2.983, Elaboration had the highest score difference between the two experiments out of all the dimensions. To be able to elaborate on something, one must first fully understand what one is talking about, which is why it is believed that this may be due to the refining of the course material making it easier for students to grasp key points, in turn making it easier for them to elaborate on the lesson content. The study can actually see this effect on the results of the other dimensions, showing that students generally performed in the second experiment; thus it was only natural for them to be able to elaborate on the topic much better with their newfound understanding of the lesson content.

The average score for the Attention dimension was 3.916, and question 2 had the highest score out of all six questions (Mean = 4.033), which shows that the majority of the students had no problem concentrating in English class. In this day and age, social media, smartphones, and other 3C products are often a source of distraction for students, which is why the old ways of teaching struggle to keep them focused on lessons. Question numbers 2, 4, 5, and 6 had a standard deviation higher than 1. It is speculated that the reasoning behind this is that some students found it difficult to focus on the lesson when paired up with their classmates, as peer interaction may sometimes be distracting. All things considered, the results of the Attention dimension still indicated that our Augmented Reality English Teaching System is beneficial for students’ learning, and is a successful example of using technology to pique students’ interest in learning, helping them focus on the class. In contrast to the first experiment where the average score was 3.234, the second experiment had a considerably higher score. This is most likely due to the pairing based on the skill level in the second experiment, which allowed for a more competitive experience for the students, in turn making them more invested in improving to beat their peers, resulting in a higher overall score for the Attention dimension.

The average score for the Peer Learning dimension was 4.040, indicating that learning with peers is an effective way of boosting students’ motivation. This score is also much higher than that of the first experiment (Mean = 3.280). It is speculated that the reason behind this is the skill-based pairing method implemented during the second experiment. By grouping students of the same level against each other, a more competitive environment was created, driving the students to perform better, an effect which can actually be seen in the Attention dimension, where results showed that students were more focused in the second experiment than in the first. All of this suggests that the 1 × 1 game mechanic in the Augmented Reality English Teaching System was quite effective in terms of helping students improve their English. However, question 1 had a lower average score than the other questions (Mean = 3.867) with a standard derivative larger than 1, meaning that only some students were able to explain the lesson content to those who did not understand. It is speculated that the reason for this was that understanding and teaching are two very different things. To properly teach others, the students have to completely understand the lesson while also being able to convey it in their own words, and sometimes they even have to find out what exactly the other student does not understand, all of which require experience and a full understanding of the topic.

These results show that there was a significant difference between each of the dimensions in the STEAM Learning Perceptions questionnaire and that using the Augmented Reality English Teaching System was considerably helpful for STEAM English education.

• What is the students’ technology acceptance of using the Augmented Reality English Teaching System?

The main purpose of the Technology Acceptance questionnaire was to determine how accepting students were of using the Augmented Reality English Teaching System in class. The questionnaire was split into two dimensions: Perceived Usefulness and Perceived Ease of Use. Perceived usefulness shows how useful students think the system is for helping them learn English, while the latter is determined by how easy it is for students to operate the system. The study used the one-sample *t*-test on the average scores of each dimension in the Technology Acceptance questionnaire for the first experiment, which was 3.000 for Perceived Usefulness, and 4.217 for Perceived Ease of Use. The results were Mean = 4.333, S.D. = 0.579, *t* = 12.623, *p*-value < 0.001, and Mean = 4.333, *S.D*. = 0.747, *t* = 0.853, *p*-value = 0.400, respectively.

According to statistics, most students’ Perceived Usefulness of the technology was quite high. The average score was 4.333, with question 2 having the highest average score of 4.400, showing us that the vast majority of students thought the Augmented Reality English Teaching System was helpful for improving their English. All other questions also had an average score higher than 4.000, which allows us to safely conclude that the use of the Augmented Reality English Teaching System could help students understand not only the class better but also key fundamentals. A high average score for question 4 also showed us that students wanted to continue using our system in their future classes. The average score in the first experiment was 3.000, much lower than in the second experiment. It is speculated that such a drastic difference was the result of changing the student pairing method and course content. Through the results of the STEAM learning perceptions questionnaire, it can be inferred that students generally had a better experience in the second experiment, and it was also more effective in helping them learn English, resulting in a higher score for Perceived Usefulness.

In the Perceived Ease of Use dimension, the average score for the first experiment was 4.217, showing that most students found operating the Augmented Reality English Teaching System to be effortless, hence our decision to keep the system as it was. The average score for Perceived Ease of Use was 4.333 the second time, quite similar to that of the first. Results for the Perceived Ease of Use dimension in Table 2 showed that every question had an average score of 4 or higher, meaning students found it easy to get used to our Augmented Reality English Teaching System. Question 4 also had a higher-than-average score, which indicates that using MR technology and a 1 × 1 game mechanic in English education is a breath of fresh air for the students, driving them to engage with the lessons and piquing their curiosity about learning.

**TABLE 2:** The result of the technology acceptance questionnaire.

Item	N	Mean	S.D.
**Perceived usefulness**			
Using the English learning app helps me better understand English.	30	4.367	0.669
When learning English, using the English learning app can get me better results.	30	4.400	0.621
The English learning app helps me understand important fundamentals of the questions I try to answer.	30	4.300	0.702
I wish to continue using this educational system when studying in the future.	30	4.267	0.828
Using the English learning app is helpful for improving my English.	30	4.333	0.758
**Perceived Ease-of-use**			
It’s easy for me to get the hang of using the English learning app.	30	4.300	0.794
I have no problem operating the English learning app independently.	30	4.333	0.884
The user interface for the English learning app is easy to use.	30	4.367	0.765
Operating the English learning app feels refreshing to me.	30	4.333	0.802

## Conclusion

Our integration of AR technology alongside a 1 × 1 core game mechanic with English education under the framework of STEAM not only solves the difficult issues for new users that Birt and Cowling (2017) faced in their research, but also has more features than Chen et al. (2018) designed in their app, allowing students to track their own progress, and allowing teachers to better focus on each individual’s weaknesses. The 1 × 1 game mode created more peer interactions, and motivated students to improve by placing them in a healthy and competitive environment, while the environment can be kept competitive by pairing students against each other based on their English proficiency. Last, by refining the course material down to common sentences and dialogue, basic tense grammar, and everyday vocabulary, this approach made it much easier for students to grasp the main focus of each unit, and apply that knowledge to real-life scenarios.

In the STEAM Learning Perceptions questionnaire, results show that after tweaking the lesson content, students had an easier time digesting what was taught in class, and could grasp the key points of each lesson, make charts to help them memorize vocabulary and grammatical rules, and not only strengthen their sense of language but boost their learning efficiency and help them apply knowledge in real life. As for the Technology Acceptance questionnaire, results show that the Augmented Reality English Teaching System is helpful for students’ learning, and is widely considered to be easy to get into and operate, which means that our study has successfully found a solution to the difficult issue that previous researchers faced. Moreover, just as presented by Chen et al. (2018), the results showed that teaching through AR technology helps increase students’ interest in and motivation for learning, and also boosts the effectiveness of their study. Similar to Chang et al. (2011), this study also shows that teaching through AR technology can draw students’ attention to learning, and it is so to speak, a potential learning tool for students. Furthermore, Lee and Hao (2013) not only show the progress of the students in learning English through AR learning materials but also indicate that teaching through daily related topics can make students link lessons to their everyday experiences.

If anyone were to embark on similar studies in the future, it would be best if the study was on a topic that the users are interested in. Take secondary school students, for example, in addition to a topic that is interesting to them, competition with each other also plays a major part in motivating students to engage in studying and other related activities. The design of the teaching aids should be faithful to the concept of “learning through entertainment.” In this study, two questionnaires were administered to gather feedback, but the information gained by this method was quite limited. If open-ended questionnaires or Q&A sessions with the students were added, it would not only verify the results of the STEAM education and technology acceptance questionnaires but also gather more rounded feedback and thoughts from the students. Moreover, the utilization of AR technology in our study still has room for improvement. Plans for the future are to more effectively use AR to connect learning with the everyday life of students, integrate their interest in AR with learning, and make peer interactions within the game more competitive.

Last but not least, we plan to add more variety to our questions in the future to make the system also be useful for writing and listening proficiency, as well as other abilities. After further improvements to our system, we will conduct another experiment, with student interviews and open-ended questionnaires at the end of it to acquire feedback and to compare to our speculations.

## Author contributions

C-CL, C-FL, T-KW, and Y-SS contributed equally to the conception of the idea, implementing and analyzing the experimental results, and writing the manuscript. All authors read and approved the final manuscript.
